# Broad-Spectrum Inhibition of the CC-Chemokine Class Improves Wound Healing and Wound Angiogenesis

**DOI:** 10.3390/ijms18010155

**Published:** 2017-01-13

**Authors:** Anisyah Ridiandries, Christina Bursill, Joanne Tan

**Affiliations:** 1 Heart Research Institute, 7 Eliza Street, Newtown, Sydney 2042, NSW, Australia; anisyah.ridiandries@hri.org.au; 2 Sydney Medical School, University of Sydney, Camperdown, Sydney 2050, NSW, Australia

**Keywords:** chemokine, angiogenesis, wound, healing, inflammation

## Abstract

Angiogenesis is involved in the inflammation and proliferation stages of wound healing, to bring inflammatory cells to the wound and provide a microvascular network to maintain new tissue formation. An excess of inflammation, however, leads to prolonged wound healing and scar formation, often resulting in unfavourable outcomes such as amputation. CC-chemokines play key roles in the promotion of inflammation and inflammatory-driven angiogenesis. Therefore, inhibition of the CC-chemokine class may improve wound healing. We aimed to determine if the broad-spectrum CC-chemokine inhibitor “35K” could accelerate wound healing in vivo in mice. In a murine wound healing model, 35K protein or phosphate buffered saline (PBS, control) were added topically daily to wounds. Cohorts of mice were assessed in the early stages (four days post-wounding) and in the later stages of wound repair (10 and 21 days post-wounding). Topical application of the 35K protein inhibited CC-chemokine expression (CCL5, CCL2) in wounds and caused enhanced blood flow recovery and wound closure in early-mid stage wounds. In addition, 35K promoted neovascularisation in the early stages of wound repair. Furthermore, 35K treated wounds had significantly lower expression of the p65 subunit of NF-κB, a key inflammatory transcription factor, and augmented wound expression of the pro-angiogenic and pro-repair cytokine TGF-β. These findings show that broad-spectrum CC-chemokine inhibition may be beneficial for the promotion of wound healing.

## 1. Introduction

Wound healing is a complex multistep process comprised of three overlapping but distinct phases: inflammation, proliferation and remodelling. It begins with the inflammation phase, where neutrophils and macrophages are recruited to the wound site to remove cell debris and phagocytose infectious pathogens. This is followed by the proliferation stage, where re-epithelialization and the formation of granulation tissue begin to close the wound. Angiogenesis, the formation of new blood vessels, is important during both the inflammation and proliferation phases, forming angiogenic capillary sprouts that allow for recruitment of inflammatory cells to the wound for debris removal, invasion of the fibrin/fibronectin-rich wound clot, and reorganization of a microvascular network to maintain the new granulation tissue being formed [1]. Finally, during the remodelling phase, fibroblasts reorganize the collagen matrix, forming a closed wound [2]. All three phases must occur in the proper sequence and time frame in order for a wound to heal successfully. Excessive macrophage accumulation during the inflammation and proliferation stages may prolong the inflammatory response leading to delayed wound healing and scar formation at the injury site. Macrophage phenotype has also been reported to change throughout the wound healing process, alternating between inflammatory M1 and repair M2 phenotypes [3]. The regulation of these phenotypes has been shown to affect wound functionality and the extent of scar tissue [4]. Wounds that exhibit impaired healing frequently enter a state of pathological inflammation, with most chronic wounds developing into ulcers. The incidence of chronic wounds is associated with ischemia, diabetes mellitus, venous stasis disease or pressure [5]. Chronic, non-healing wounds greatly impact a person’s quality of life and, when left untreated, sometimes lead to amputation, resulting in an enormous health care burden [6,7]. While current therapies provide some relief, there is a need for the continued development of novel therapies that address the debilitating effects of impaired wound healing.

CC-chemokines are small inflammatory cytokines that have been shown to play a key role in the induction of inflammation and inflammation-mediated angiogenesis. In the wound healing process, macrophage infiltration is highly regulated by CC-chemokine gradients released by hyper-proliferating keratinocytes, fibroblasts and other macrophages [8,9]. In human wounds, a host of CC-chemokines including CCL1, CCL2, CCL3, CCL4, CCL5 and CCL7 are expressed during the first week after injury and high levels of CCL2 have been found in human burns [10,11]. Additionally, studies in excisional wounds have found localized expression of CCL2 and CCL3 in the epidermis, while CCL3 is expressed in follicular epithelium and sebaceous glands [12]. CC-chemokines regulate key angiogenic processes such as the recruitment of inflammatory cells to the wound, to provide support for proliferating and migrating cells [13], and the formation of granulation tissue [9]. Furthermore, incubation with the recombinant CC-chemokines CCL2 and CCL5 stimulate angiogenic functions such as endothelial cell tubulogenesis and migration [14,15,16,17,18]. Therefore, manipulation of the CC-chemokine family may modulate key wound healing processes to confer benefits. To date, studies have inhibited a single CC-chemokine and shown modest or no effect on wound healing. CCL2 knockout mice have delayed re-epithelialization and reduced angiogenesis in the early stages of wound repair, whilst the CCL3 knockout mice have normal wound healing [19]. Similarly, CCR1 knockout models have no alteration in wound healing [20]. Inhibition of a single CC-chemokine, however, may not be as effective in modulating wound healing due to redundancies in CC-chemokine signalling [21,22]. A broad-spectrum CC-chemokine inhibition approach may therefore have increased efficacy.

The broad-spectrum CC-chemokine inhibitor “35K” is a 35 kDa soluble protein produced by the Vaccinia virus that uniquely inhibits only the CC-chemokine class [23]. It recognizes and binds to common structural features shared by most CC-chemokines, preventing binding to their cognate receptors [24]. Broad-spectrum CC-chemokine inhibition using 35K inhibits a host of inflammatory diseases including atherosclerosis, acute peritonitis, hepatitis and liver fibrosis [25,26,27,28]. Given the inhibitory effects of 35K on inflammatory-mediated diseases and the role of CC-chemokines in angiogenesis, we sought to elucidate the effect of broad-spectrum CC-chemokine inhibition on wound healing. We found that topical application of 35K enhanced wound closure, blood flow perfusion and neovascularisation at the early-mid stages of wound healing. Augmentation of wound angiogenesis by 35K was mediated via an increase in the pro-angiogenic and pro-repair cytokine TGF-β. In addition, 35K treatment inhibited wound levels of inflammatory transcription factor NF-κB and reduced collagen deposition at the late stages of wound healing in the 35K-treated wounds, suggesting a reduction in scar formation. Taken together, these findings show that broad-spectrum inhibition of CC-chemokines may be beneficial for the promotion of wound healing by suppressing inflammation and promoting wound closure and neovascularisation and reducing scar formation.

## 2. Results

### 2.1. Topical 35K Increases Wound Blood Perfusion and the Rate of Wound Closure in Early-Mid Stage Wound Repair

**Figure 1:**
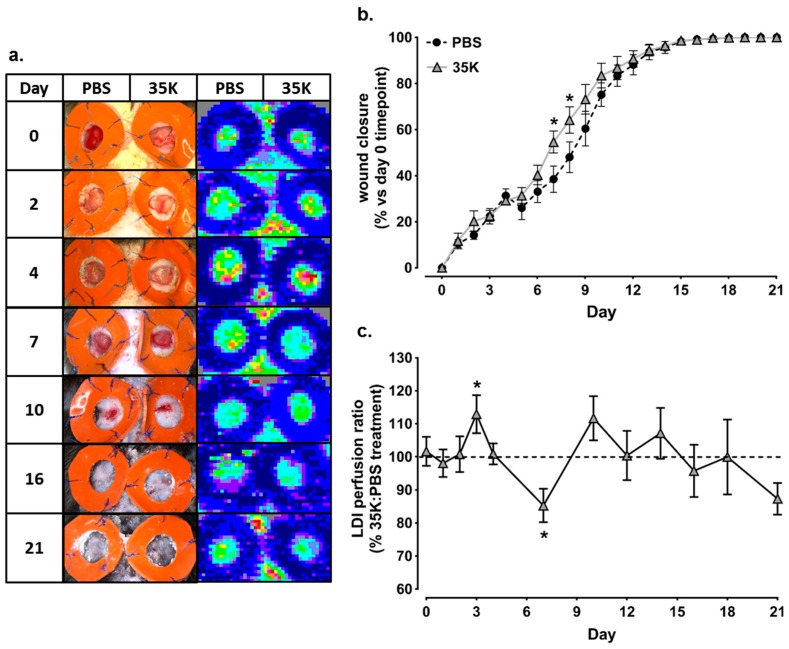
Topical 35K increases the rate of wound closure and wound blood perfusion in early-mid stage wound repair. Two full thickness wounds were created on C57Bl/6J mice (*n* = 7–12). Mice received daily topical application of 35K protein (200 nM) or PBS (vehicle). (**a**) representative images of the wounds and wound blood perfusion using Laser Doppler imaging (high (red) to low (blue) blood flow); (**b**) wound area was calculated from the average of three daily diameter measurements along the *x*-, *y*- and *z*-axes. Wound closure is expressed as a percentage of initial wound area at Day 0. Black circles are PBS treated wounds; grey triangles are 35K treated wounds; and (**c**) the 35K:PBS wound blood flow perfusion ratio was determined using Laser Doppler imaging (LDI). Data is represented as mean ± SEM. Points above the dotted line represent an improvement with 35K treatment. Statistical analysis was performed by an unpaired two-tailed *t*-test. * *p* < 0.05 compared to PBS treated wounds.

Topical treatment of wounds with 35K protein caused significantly faster wound closure throughout the early to mid-stages of wound healing at Day 7 (42%, *p* < 0.05) and Day 8 (33%, *p* < 0.05) compared to PBS treated wounds (Figure 1a). Using Laser Doppler imaging, it was revealed that 35K protein augmented wound blood perfusion (Figure 1b). The Laser Doppler Index (LDI), used as a marker of wound angiogenesis and determined as the ratio of 35K:PBS wounds, was significantly elevated in the 35K treated wounds in the early stages following wounding (Day 3, 4), compared to PBS control wounds (values above 100% indicate increased wound blood perfusion with 35K treatment relative to PBS control). In the later stages of the wound healing process, at seven days post-wounding, blood perfusion started to decline in wounds treated with 35K, compared to the PBS treated control wounds. After Day 7, blood perfusion fluctuated along the baseline and very little blood perfusion was detected. There were no significant increases or decreases to the end point.

### 2.2. Inhibition of CC-Chemokines by 35K Increases Neovessel Formation in Early Stage Wounds but Decreases Neovessels in Late Stage Wounds

**Figure 2:**
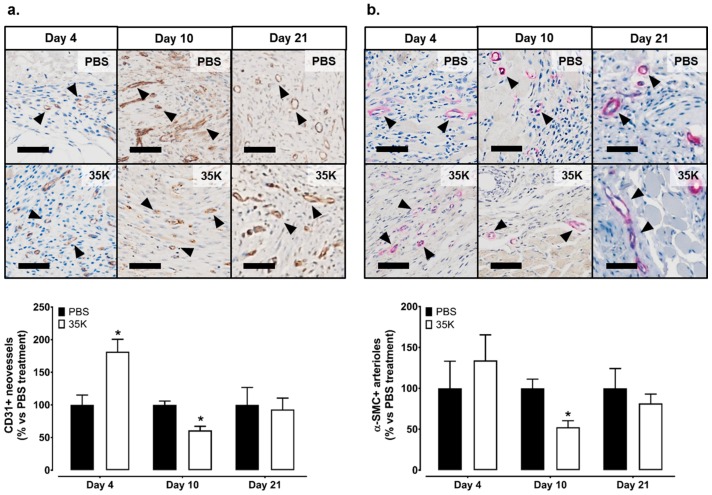
Inhibition of CC-chemokines by 35K increases neovessel formation in the early stages of wound repair but decreases neovessels in later stages. Immunocytochemistry was used to detect the presence of neovessels and arterioles. Photomicrographs represent wounds stained for (**a**) CD31+ neovessels (stained brown, noted by black arrows) and (**b**) α-SMC+ arterioles (stained pink, noted by black arrows). Three tiled images were taken per wound (*n* = 7–12/treatment). Scale bars represent 50 μm. Data is represented as mean ± SEM. Statistical analysis was performed by an unpaired two-tailed *t*-test. * *p* < 0.05 compared to PBS treated wounds.

To investigate wound angiogenesis, neovessels and arterioles were assessed. In the early stages of wound healing (Day 4), there was an increase in the presence of wound neovessels in the 35K treated wounds as determined by CD31+ staining (182%, *p* < 0.05, Figure 2a). However, at Day 10 post-wounding, there were significantly fewer wound neovessels (39% decrease, *p* < 0.05) following 35K treatment. At Day 21, there were no differences in neovessels between 35K and PBS treated wounds. A similar biphasic pattern was also seen with the arterioles (α-actin+ staining), although, at Day 4, the trend for an increase in arterioles did not reach significance, but there was a significant 48% decrease in arterioles (*p* < 0.05) in the 35K treated wounds at Day 10 post-wounding (Figure 2b). No CD31+ and α-actin+ staining is detected in IgG controls (Figure S1).

### 2.3. Inhibition of CC-Chemokines by 35K Modulates Pro-Angiogenic Markers TGF-β and Vascular Endothelial Growth Factor (VEGF) in Early and Late Stage Wounds

**Figure 3:**
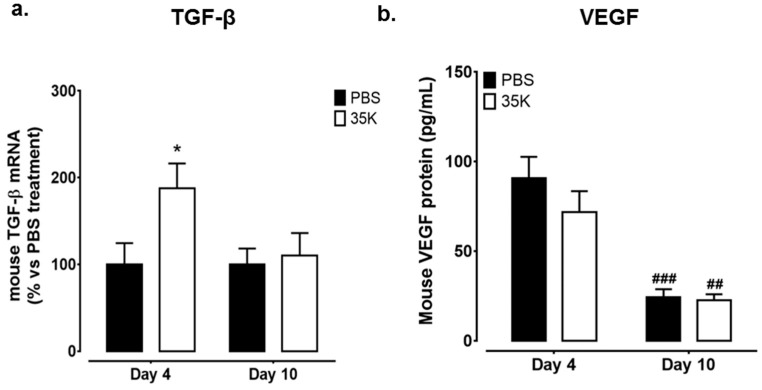
Modulation of pro-angiogenic markers following inhibition of CC-chemokines by 35K in early and late stage wounds. RNA and protein were isolated from PBS and 35K treated wounds at both the early (Day 4) and late (Day 10) time points (*n* = 12/time point); (**a**) real-time PCR was used to measure mRNA levels of TGF-β; (**b**) vascular endothelial growth factor (VEGF) protein levels were detected by ELISA. Data is represented as mean ± SEM. Statistical analysis was performed by an unpaired two-tailed *t*-test. ## *p* < 0.01, ### *p* < 0.001 compared to respective treatment group wounds at Day 4. * *p* < 0.05 compared to PBS treated wounds.

The expression of key angiogenic markers TGF-β and vascular endothelial growth factor (VEGF), known to be involved in wound healing, were measured next. Interestingly, TGF-β expression was significantly higher in 35K-treated wounds in the early stages of wound repair (Day 4 post-wounding, 88%, *p* < 0.05, Figure 3a). However, in the later stages post-wounding (Day 10), the expression of TGF-β had normalised back to control levels. There were no differences in VEGF protein levels at Day 4 post-wounding between treatment groups. In the later stages, wounds from both treatment groups had significantly lower levels of VEGF protein, compared to wounds at Day 10 (*p* < 0.01). The protein levels of fibroblast growth factor-2 (FGF-2), were also measured, but no differences were detected (Figure S2a). Neither were there changes in wound protein levels of HIF-1α, a regulator of VEGF (Figure S2b).

### 2.4. Inhibition of CC-Chemokines by 35K Reduces Inflammation but Has No Effect on Wound Macrophage Content

**Figure 4:**
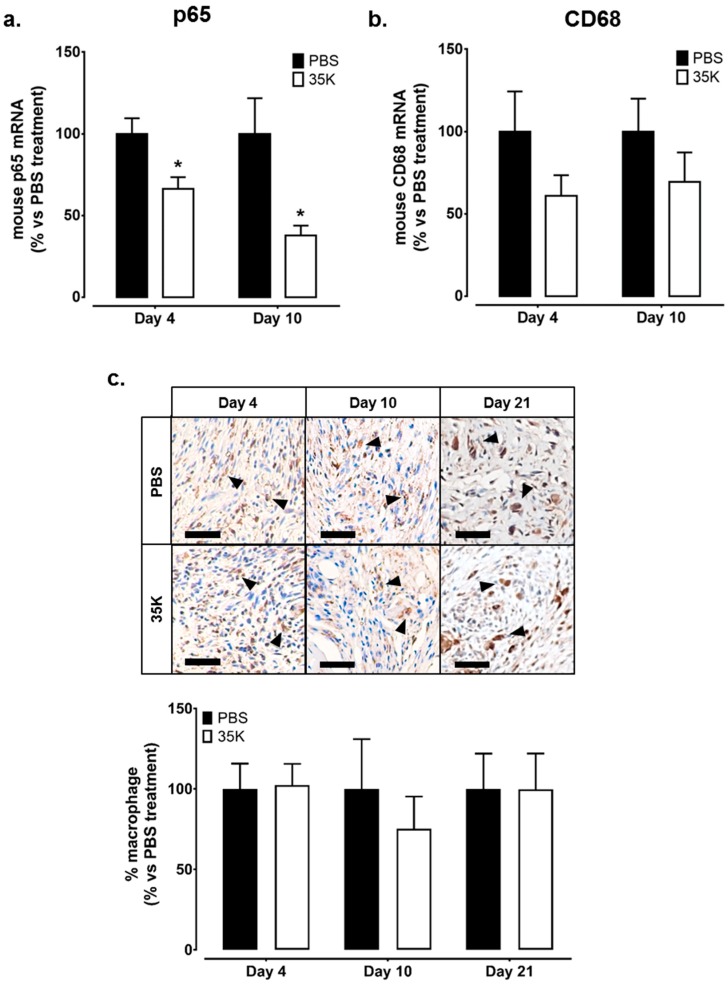
Inhibition of CC-chemokines by 35K reduces inflammation but has no effect on wound macrophage content. Total RNA was isolated from PBS and 35K treated wounds at both the early (Day 4) and late (Day 10) time points (*n* = 12/time point). Real-time PCR was used to measure mRNA levels of (**a**) p65, the active subunit of the key inflammatory transcription factor NF-κB and (**b**) macrophage marker CD68; (**c**) immuno-histochemistry was used to detect the presence of wound macrophages. Photomicrographs represent wound sections stained for CD68+ macrophages (stained brown, noted by black arrows). Three tiled images were taken per wound (*n* = 7–12/treatment). Scale bars represent 50 μm. Data is represented as mean ± SEM. Statistical analysis was performed by an unpaired two-tailed *t*-test. * *p* < 0.05 compared to PBS treated wounds.

Broad-spectrum CC-chemokine inhibition suppressed the mRNA levels of p65, the active subunit of NF-κB, with 34% and 62% decreases seen in Day 4 and 10 in 35K treated wounds respectively, compared to PBS treated wounds (all *p* < 0.05, Figure 4a). A similar trend was observed for mRNA levels of the macrophage marker CD68, which were also lower in the 35K treated wounds but did not reach significance (Day 4: 39% ns; Day 10: 30% ns, Figure 4b). Despite this, histological analysis of macrophages in wound sections did not find a difference in CD68+ cells in 35K and PBS treated wounds at Day 4, 10, and 21 post-wounding (Figure 4c). Analysis of wound macrophage phenotype revealed that there were no significant changes in the mRNA levels of M2 macrophage marker CD206 in the 35K treated wounds (Figure S3a). Interestingly, M1 macrophage markers CD80 and CD86 were unchanged at Day 4 but elevated in Day 10 35K treated wounds (278% and 342% respectively, *p* < 0.01, Figure S3b,c). No CD68+ staining is detected in IgG control (Figure S1).

### 2.5. Inhibition of CC-Chemokines by 35K Decreases Collagen Formation in Late Stage Wounds

**Figure 5:**
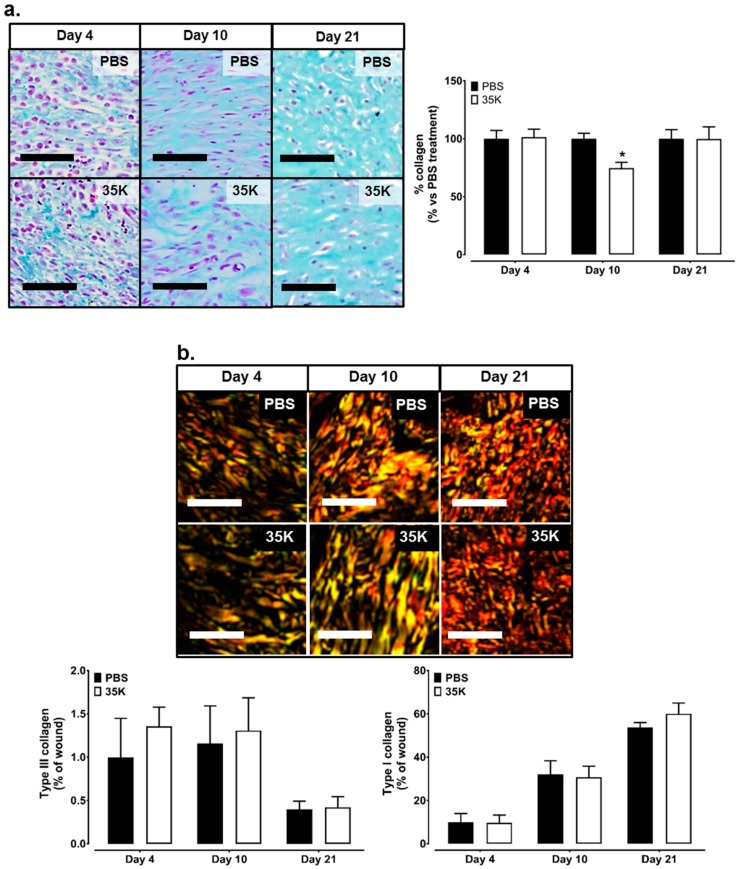
Inhibition of CC-chemokines by 35K decreases collagen formation in Day 10 wounds. Masson’s trichrome staining was used to detect the collagen content in the wounds. (**a**) green staining in wound sections is collagen, purple staining are nuclei; (**b**) to differentiate collagen type, wound sections were stained red with picrosirius and imaged under polarized light. Green represents Type III collagen, yellow to red represents Type I collagen. Three tiled images were taken per wound (*n* = 12 or 7/treatment). Scale bars represent 50 μm. Data is represented as mean ± SEM. Statistical analysis was performed by an unpaired two-tailed *t*-test. * *p* < 0.05 compared to PBS treated wounds.

Milligan’s trichrome staining was used to detect the collagen content in the wounds as a marker of tissue remodelling. While no differences in collagen deposition were observed at Day 4 and 21 between 35K and PBS wounds, a 25% decrease (*p* < 0.05) in collagen content was seen in the 35K treated wounds at Day 10 (Figure 5a). The picrosirius red stain was imaged under polarized light to differentiate between type III and type I collagen. There was 10–100 times more type I collagen to type III collagen (across Day 4–21), but there were no significant differences between 35K and PBS treated wounds at each time point (Figure 5b).

### 2.6. Topical 35K Suppresses CC-Chemokine Expression in the Wounds

**Figure 6:**
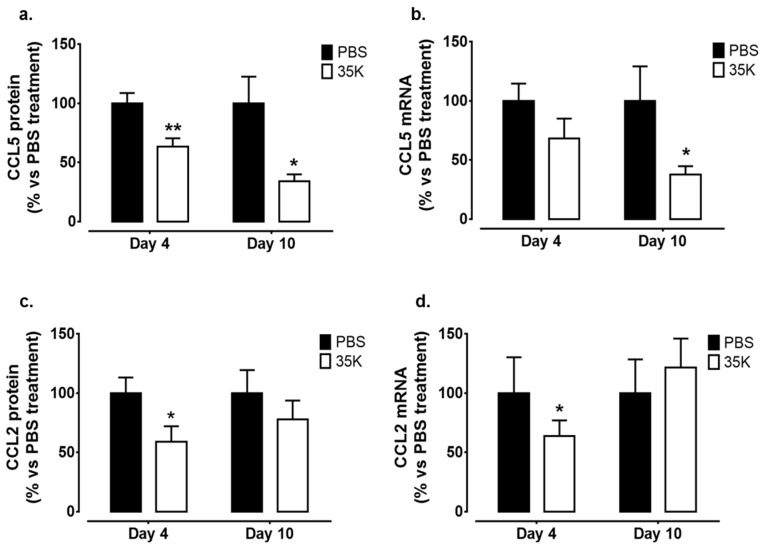
Topical 35K suppresses CC-chemokines in wound tissues. Protein and RNA were isolated from PBS and 35K treated wounds at both the early (Day 4) and late (Day 10) time points (*n* = 12/time point). ELISAs and real-time PCR was used to measure protein and mRNA levels of (**a**,**b**) CCL5 and (**c**,**d**) CCL2. Data is represented as mean ± SEM. Statistical analysis was performed by an unpaired two-tailed t-test. * *p* < 0.05, ** *p* < 0.01 compared to PBS treated wounds.

The effect of 35K on wound CC-chemokine protein and gene expression was measured. There was a significant decrease in CCL5 protein levels in 35K treated wounds at both the Day 4 (36%, *p* < 0.05) and Day 10 (66%, *p* < 0.05) time points (Figure 6a). There were also similar decreases in CCL5 mRNA levels in wounds treated with 35K (Day 4: 32%, ns and Day 10: 62%, *p* < 0.05, Figure 6b). CCL2 protein levels were lower in wounds at the Day 4 time point (41%, *p* < 0.05, Figure 6c), but the trend for a decline at the Day 10 time point did not reach significance following 35K treatment. This was consistent for wound CCL2 mRNA levels in which 35K treatment decreased CCL2 wound mRNA at the Day 4 time point (36%, *p* < 0.05, Figure 6d), with no significant decrease in Day 10 wounds.

## 3. Discussion

The stages of the wound healing process encompass the most complex biological processes that occur in human life. An imbalance can greatly affect the outcome of wound repair, with impaired wound healing often leading to severe unfavourable outcomes such as amputation. This highlights the need to develop novel agents that promote wound recovery. CC-chemokines have been shown to play a key role in the promotion of inflammation and inflammatory-induced angiogenesis, two important processes involved in wound healing. To date, only single CC-chemokine intervention studies have been done, with minimal to no benefit, suggesting that a broad-spectrum inhibition approach may have improved efficacy. In this study, we report that topical application of the broad-spectrum CC-chemokine inhibitor “35K” decreased wound CCL5 and CCL2 protein levels, leading to enhanced wound healing and wound neovascularisation during the early stages of wound healing. Histological analysis of wounds showed that 35K treatment significantly increased neovessels in the early stages of wound healing, when angiogenesis is most important. Mechanistically, we found that the classical VEGF-mediated angiogenic pathway was not responsible for the augmentation of angiogenesis by 35K, but, instead, 35K elevated the pro-angiogenic and pro-repair cytokine TGF-β. Furthermore, 35K treated wounds had significantly lower mRNA levels of p65, the active subunit of the inflammatory transcription factor NF-κB. In addition, 35K treated wounds at Day 10 had lower collagen content, suggesting a reduction in scar formation. Taken together, these findings suggest that broad-spectrum inhibition of CC-chemokines may be an alternate therapeutic approach to improve wound healing by simultaneously suppressing inflammation while augmenting neovascularisation at the critical early stages of wound repair.

Macroscopic wound measurements revealed that wound closure was faster in 35K treated wounds compared to PBS treated control wounds. This was supported by the increased blood flow perfusion observed during the early stages post-wounding in 35K treated wounds. At this stage (~Day 3), perfusion is critical to support the wound healing process to supply the wound with nutrients and growth factors, and accelerate debris removal [2,29]. Increased blood flow perfusion also allows for increased oxygenation to help maintain the newly forming wound tissue [30]. This early boost in perfusion may support the increased closure seen at Day 7 and 8. By Day 7, wound blood perfusion then started to decline in the 35K treated wounds. At this stage, remodelling is commencing, and, whilst neovessels are still present, the impetus for new wound neovessels has started to decline, supporting the decrease in wound blood perfusion at this stage. After Day 7 post-wounding, there is very little detectable blood perfusion using the Laser Doppler for both treatments. Consistent with our blood perfusion measurements, the current study also found that topical application of 35K increased the presence of wound neovessels at Day 4, and, at Day 10, neovessels and arterioles were reduced in the 35K treated wounds. At this late stage, the wound has nearly healed, re-epithelialization is complete, inflammation has ceased, a scar has formed and collagen remodelling from type III to type I collagen begins [31]. Importantly, as the wound healing process is near-completion, angiogenesis may decline to allow for continued remodelling [9,32]. The lower number of neovessels and arterioles seen at Day 10 suggest that these wounds have healed quicker and have now entered the remodelling phase. However, at Day 21, the wounds are further in the remodelling phase, bringing stability to neovessel content. The reduction in collagen content of the 35K treated wounds at Day 10 may further lead to reduced scar formation, as scars are mainly comprised of collagen [33]. At Day 21, the collagen content returns to a stable level, when compared to PBS wounds. This suggests that whilst the wounds may have less scar the stability of the skin is not compromised. Interestingly, at Day 4 there is 10 times more type I collagen than type III, and, when the wound is completely healed and undergoing remodelling, there is an increase of 50 times in type I collagen in wounds. These findings are consistent with previous studies where reduced fibrosis was detected in the kidneys and lungs of mice following inhibition of the chemokine receptors CCR1 or CCR5 [34,35,36].

Angiogenic mediators such as VEGF and FGF-2 are released by various cell types including endothelial cells, fibroblasts, keratinocytes and macrophages. VEGF is primarily produced by endothelial cells, whilst FGF-2 is produced by fibroblasts. Interestingly, 35K had no effect on these key angiogenic mediators involved in wound healing. Furthermore, we found no change in wound HIF-1α expression, which regulates VEGF and is triggered in response to tissue ischemia. This suggests that there was sufficient blood perfusion and oxygenation to the wounds. VEGF is involved in angiogenesis and synthesis of collagen in the wound [8,37]. It is produced by wound fibroblasts, keratinocytes and macrophages [8,32,38]. VEGF levels remained unchanged in 35K treated wounds, suggesting that VEGF is not involved in the augmentation of wound healing and wound angiogenesis by 35K. An alternative suggestion is that VEGF expression increased very early post-wounding with 35K, which was missed at the Day 4 time point. Studies have shown increases in VEGF just 24 h post-wounding [39], which then return to baseline once angiogenesis is underway. There was, however, an increase in wound neovessels with 35K treatment at the Day 4 time point, despite no changes in VEGF or HIF-1α. Interestingly, both VEGF and HIF-1α are induced by NF-κB in inflammatory pathological angiogenesis. This study found that 35K significantly inhibited p65 (active NF-κB subunit) mRNA levels. This reduction in NF-κB may be part of the reason for the lack of change in VEGF and HIF-1α and suggests that 35K was preventing pathological excessive wound angiogenesis. Furthermore, the reduction in wound CC-chemokine levels by 35K would also contribute to the suppression of pathological angiogenesis that can compromise wound repair if it persists in the later stages of healing. The increase in angiogenesis seen during early wound healing, where it is critical, is likely to be the result of increased TGF-β expression in the Day 4 35K treated wounds. TGF-β increases key angiogenic functions including endothelial cell proliferation and migration [40,41]. TGF-β also has a number of roles in the wound repair process including fibroblast proliferation, collagen formation and remodelling of the extracellular matrix [42] and the initiation in the formation of granulation tissue [43]. TGF-β stimulates contraction of fibroblasts [44] and promotes the migration of keratinocytes in the wound [45] which promotes wound closure. Taken together, our results demonstrate that 35K does not augment wound angiogenesis via the classical HIF-1α/VEGF angiogenic pathway but rather through the induction of TGF-β, and may in fact suppress excessive pathological angiogenesis in the mid-late stages of wound repair via inhibition of NF-κB and CC-chemokine levels.

Despite the reduction in NF-κB by 35K, there were no changes in the number of wound macrophages as may have been expected. Macrophages can be roughly divided into pro- and anti-inflammatory phenotypes (M1 and M2) and both are involved in different stages of wound healing [46]. Our results indicate an increase in the M2 macrophage phenotype during the early wound healing stage (TGF-β, CD206), which is known to be associated with improved wound repair, sufficient formation of granulation tissue and new vascular networks [46]. Interestingly, there was a surprising increase in M1 macrophage markers at Day 10 (CD80, CD86), which is inconsistent with the reduction in NF-κB at this time point. However, previous studies have shown that the presence of M1 macrophages at this late-stage does not affect the wound healing process [4].

There was a decrease in CCL5 and CCL2 protein in the tissues of wounds treated with the CC-chemokine inhibitor 35K. This would typically be associated with a decrease in macrophages; however, no significant reductions in macrophages were observed. A previous study reported that despite dominant expression of CCL2 in dermal mouse wounds, a relatively low CCL2 level correlated with optimal monocyte accumulation [12], indicating that a reduction, but not complete ablation of CCL2, may not affect macrophage recruitment in the early stages of wound healing. However, at the later stage (i.e., Day 10), the wound is preparing to enter the remodelling phase in which macrophages undergo apoptosis and chemokines and angiogenesis are subsequently reduced. Additionally, chemokines from other classes and other cytokines that are not affected by 35K (for example CX_3_CL1, IL-1, IFN-γ) are released from the wound site and may have assisted in maintaining macrophage infiltration [47], even with lower CCL2 and CCL5 levels. This may explain the consistent macrophage levels seen in our study despite the reduction in CC-chemokine levels.

## 4. Materials and Methods

### 4.1. Generation and Isolation of 35K Protein

A recombinant adenovirus overexpressing 35K (Ad35K) was generated as described previously [48]. To isolate 35K protein, 20 large flasks of Ad293 cells (293AD cell line, AD-100, Cell Biolabs Inc., San Diego, CA USA) were infected with 5 × 10^11^ Ad35K virus particles. After 24–48 h, cells were microscopically examined for evidence of complete cytopathic effect (CPE). 35K protein was isolated from Ad35K viral media using anti-HA tagged agarose-conjugated beads (A2095, Sigma-Aldrich, St. Louis, MO, USA). The media was run through a column packed with anti-HA agarose beads to bind 35K protein, which was then eluted from the column with 3 M sodium thiocyanate into 1 M Trizma Base (pH 8). Isolated 35K protein was dialyzed into sterile PBS and then filtered through 0.45 μm low protein binding syringe filter (Pall Corporation, Port Washington, NY, USA). The concentration of 35K protein was determined by measuring absorbance at 280 nm and aliquoted to 200 nM before freezing at −80 °C.

### 4.2. Murine Wound Healing Model

All experimental procedures and protocols were conducted with approval from the Sydney Local Health District Animal Welfare Committee (#2013/027A), and conformed to the Guide for the Care and Use of Laboratory Animals (United States National Institute of Health, Bethesda, MD, USA). To explore the effect of broad-spectrum CC-chemokine inhibition on wound healing, a murine model that closely mimics the human wound healing process was used [49]. Briefly, C57Bl6/J wildtype mice were anesthetized by inhalation of methoxyflurane. Then, a 6 mm biopsy punch was used to outline two circular full-thickness excisions, 5 mm in diameter, that include the panniculus carnosus created on the dorsum, one on each side of the midline of the mouse. A 0.5 mm thick silicone splint (Life Technologies, Carlsbad, CA, USA) was then placed around the wound and secured with interrupted sutures. For each mouse, one wound received purified 35K protein (200 nM in 50 μL PBS) and the other endotoxin-free PBS (50 μL, vehicle control), topically applied daily beginning on the day of wounding (Day 0). A transparent occlusive dressing (Opsite™ Flexifix™, Smith & Nephew, London, UK) was then applied. Mice were given carprofen (5 mg/kg) daily 3 days post-surgery to alleviate any pain. Digital images were taken and micro-callipers were used to measure wound area daily along the *x*-, *y*- and *z*-axes. Blood perfusion in wound areas was determined using Laser Doppler Perfusion Imaging (moorLDI2-IR, Moor Instruments, Devon, UK). For this study, two cohorts were taken at 4 (*n* = 12), 10 (*n* = 12), or 21 (*n* = 7) days after wounding. Both PBS and 35K treated wounds were collected for histological, protein and gene analysis.

### 4.3. Immunohistochemistry

Wound tissues were fixed in 4% (*v*/*v*) paraformaldehyde overnight, and then embedded in paraffin. Furthermore, 5 µm wound sections were taken from the midpoint. Wounds were assessed for neovessels by detection with rabbit polyclonal anti-CD31 antibody (1:100, ab28364, Abcam, Cambridge, UK), arterioles by detection with mouse monoclonal smooth muscle α-actin antibody (1:100, A5691, Sigma-Aldrich) and for macrophages by detection with mouse monoclonal anti-CD68 antibody (1:100, ab31630, Abcam) staining. Secondary antibodies were pre-diluted horseradish peroxidase (HRP) secondary antibody α-rabbit (K4011, Dako) or pre-diluted HRP secondary antibody α-mouse (K4007, Dako, Glostrup, Denmark) for CD31 and CD68, respectively. The staining was visualized using 3,3′-Diaminobenzidine (DAB) (Dako) for CD31 and CD68 or Vector Red alkaline phosphatase substrate (SK-5100, Vector Laboratories, Burlingame, CA, USA) for smooth muscle α-actin. Appropriate IgG controls were used for all antibodies. Collagen was measured following Milligan’s trichrome staining. Picrosirius red staining, imaged under polarized light, was used to differentiate type I and type III collagen. For all histological quantification, three sections were imaged at 10× magnification, the images were tiled and “stitched” to obtain the entire wound cross section for each treatment group per mouse (*n* = 7–12) and assessed using Image-Pro^®^ Premier 9.0 software (Media Cybernetics, Rockville, MD, USA).

### 4.4. Protein Expression

Wound tissues were homogenized in lysis buffer (80 mM Tris HCl, 10 mM NaCl, 50 mM NaF, 5 mM Na_4_P_2_O_7_, 15 mM Triton-X 100). Wound tissue lysates (50 μg) were subjected to Western immunoblotting and probed for HIF-1α (1:1000, NB100-105, Novus Biologicals, Littleton, CO, USA) α Tubulin (1:5000, AB40742, Abcam) was used to confirm even protein loading. Commercially available ELISA kits (Quantikine, RnD Systems, Minneapolis, MN, USA) were used to determine protein levels of VEGF, CCL2 (MCP-1) and CCL5 (RANTES) in 100 μg of wound tissue lysates, while FGF-2 protein levels were measured in 50 μg of wound tissue lysates.

### 4.5. Gene Expression

Total RNA was isolated from wound samples using TRI reagent (Sigma-Aldrich). Furthermore, 300 ng total RNA was reverse transcribed using the iScript cDNA synthesis kit (Bio-Rad, Hercules, CA, USA) before amplification using the iQ SYBR Supermix (Bio-Rad) in a Bio-Rad Cfx384 thermocycler. The following mouse primers were used to probe for HIF-1α (F 5′-TCCCTTGCTCTTTGTGGTTGGGT-3′, R 5′-AACGTAAGCGCTGACCCAGG-3′), VEGF (F 5′-GAGTACCCCGACGAGATAGAGT-3′, R 5′-GGTGAGGTTTGATCCGCATGA-3′), p65 (F 5′-AGTATCCATAGCTTCCAGAACC-3′, R 5′-ACTGCATTCAAGTCATAGTCC-3′), CD68 (F 5′-GGGGCTCTTGGGAACTACAC-3′, R 5′-GTACCGTCACAACCTCCCTG-3′), TGF-β (F 5′-GGATACCAACTATTGCTTCAGCTCC-3′, R 5′-AGGCTCCAAATATAGGGGCAGGGTC-3′), CD206 (F 5′-CAGGTGTGGGCTCAGGTAGT-3′, R 5′-TGTGGTGAGCTGAAAGGTGA-3′), CD80 (F 5′-ACCCCCAACATAACTGAGTCT-3′, R 5′-TTCCAACCAAGAGAAGCGAGG-3′), CD86 (F 5′-CAGCTCACTCAGGCTTATGTTT-3′, R 5′-TGTTTCCGTGGAGACGCAAG-3′), CCL2 (F 5′-GCTGGAGCATCCACGTGTT-3′, R 5′-ATCTTGCTGGTGAATGAGTAGCA-3′), CCL5 (F 5′-GCAAGTGCTCCAATCTTGCA-3′, R 5′-CTTCTCTGGGTTGGCACACA-3′) and 36B4 (F 5′-CAACGCAGCATTTATAACCC-3′, R 5′-CCCATTGATGATGGAGTGTGG-3′). Relative changes in gene expression were normalized using the ΔΔ*C*_t_ method to 36B4 as the housekeeping gene.

### 4.6. Statistics

All results are expressed as mean ± SEM. All data were compared using an unpaired two-tailed *t*-test. Significance was set at a value of *p* < 0.05.

## 5. Conclusions

**Figure 7:**
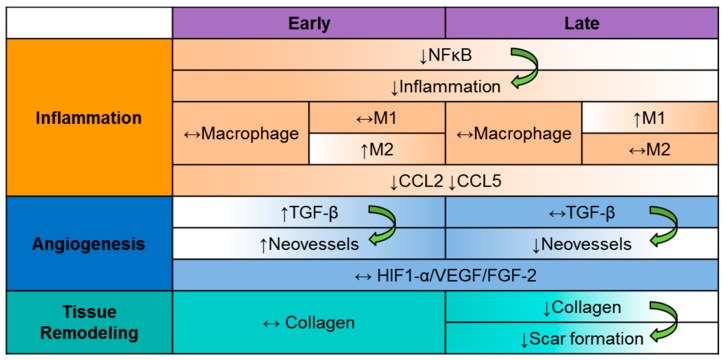
Summary of proposed 35K action in wound healing. Broad-spectrum CC-chemokine inhibition by 35K has a significant impact on key mechanisms involved in wound healing. In addition, 35K enhances wound closure through the early promotion of neovascularisation via the induction of TGF-β, rather than the HIF-1α/VEGF angiogenic pathway. 35K treatment reduces inflammation via inhibition of NF-κB, which may help to stave off pathological angiogenesis in the later stages of wound repair, along with the reductions in CCL5 and CCL2 but with no change in macrophages. Finally, 35K suppresses collagen deposition at the late stage of wound healing, but not early, indicative of reduced scar formation.

In conclusion, our findings show that broad-spectrum CC-chemokine inhibition via topical application of 35K enhanced wound closure through the early promotion of neovascularisation. Furthermore, inhibiting the CC-chemokine class resulted in a reduced inflammatory state as indicated by reduced NF-κB. Mechanistically, 35K augmented wound angiogenesis via the induction of the pro-angiogenic cytokine TGF-β, rather than the HIF-1α/VEGF angiogenic pathway. Taken together, our findings (summarized in Figure 7) demonstrate that broad-spectrum CC-chemokine inhibition may improve wound healing by suppressing the inflammatory state of the wound while augmenting wound angiogenesis and suppressing scar formation.

## Supplementary Materials

Supplementary materials can be found at www.mdpi.com/1422-0067/18/1/155/s1.
