# Supplementary material for: Broad-Spectrum Inhibition of the CC-Chemokine Class Improves Wound Healing and Wound Angiogenesis
Source: Int J Mol Sci. 2017 Jan 13;18(1):155. doi: 10.3390/ijms18010155 (PMC5297788; doi:10.3390/ijms18010155)
Supplement: Supplementary file 1 [file ijms-18-00155-s001.pdf]

Supplementary Materials: Broad-Spectrum Inhibition of the CC-Chemokine Class Improves Wound Healing and Wound Angiogenesis

Anisyah Ridiandries, Christina Bursill and Joanne Tan

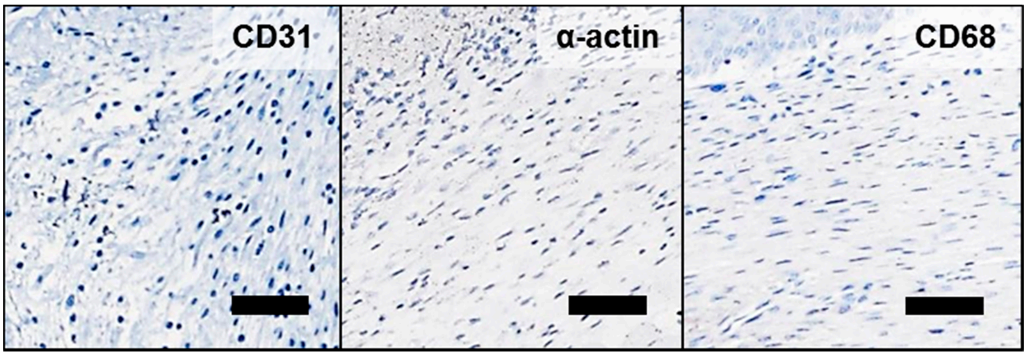

Figure S1. Cutaneous wounds from the wound healing model show negative IgG staining for CD31, smooth muscle α-actin, and CD68. Scale bars represent 50 μm.

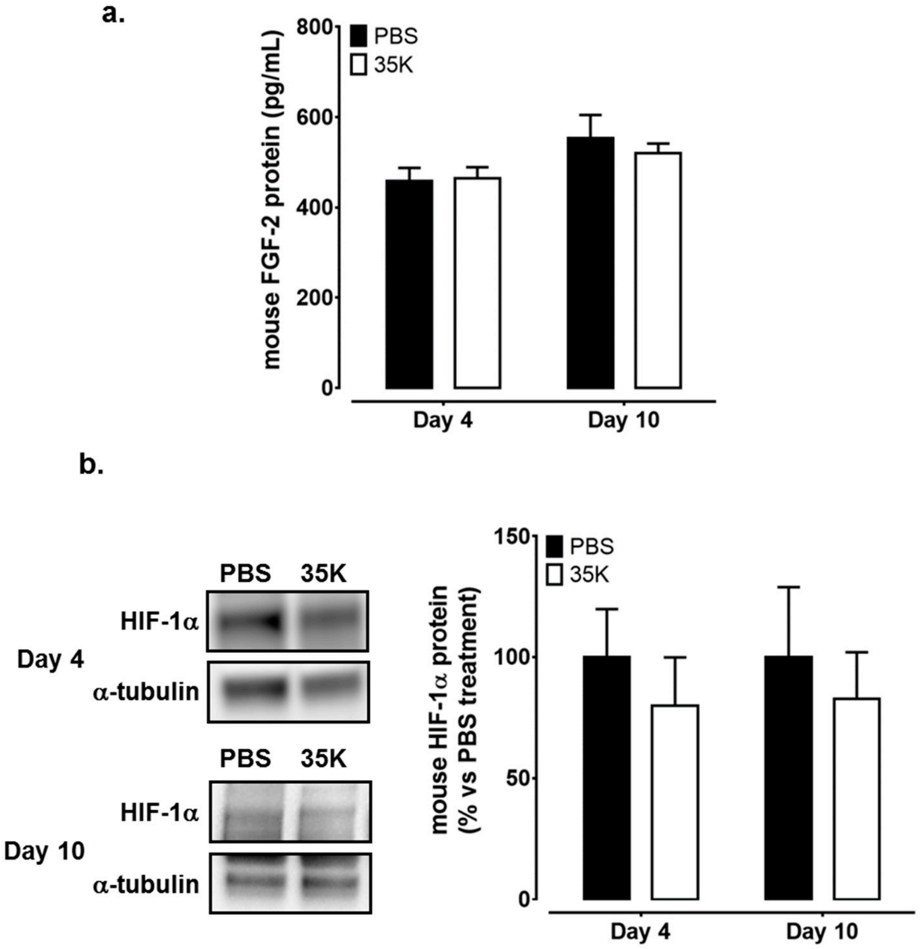

Figure S2. Regulation of wound FGF and HIF-1 α protein levels by 35K. Protein lysates were prepared from PBS and 35K treated wounds at both the early (Day 4) and late (Day 10) time points (n = 12/time point). (**a**) ELISAs were used to determine FGF proteins levels and (**b**) Western blotting was used to assess HIF-1α protein levels, with even loading confirmed by α-tubulin. Data is represented as mean ± SEM.

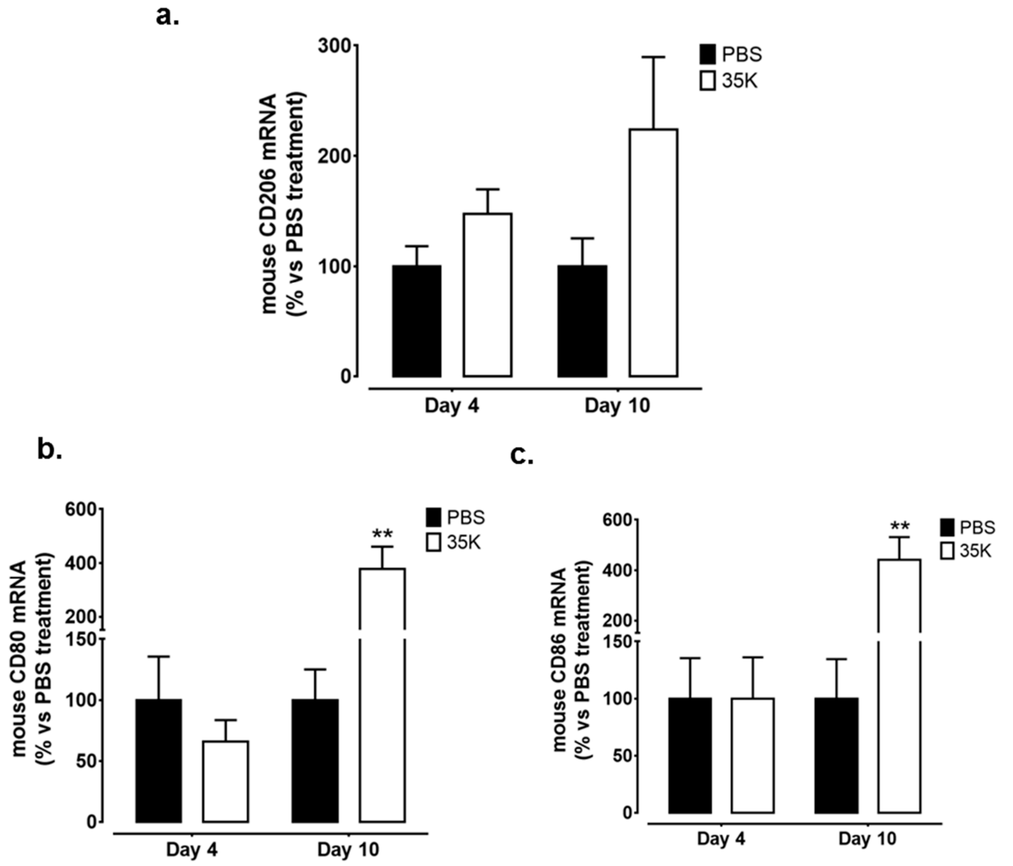

Figure S3. Regulation of M2 and M1 macrophage phenotype markers in wounds by 35K. Total RNA was isolated from PBS and 35K treated wounds at both the early (Day 4) and late (Day 10) time points ($n = 12$/time point). Real-time PCR was used to measure mRNA levels of (**a**) M2 marker CD206 and M1 markers (**b**) CD80 and (**c**) CD86. Data is represented as mean ± SEM. Statistical analysis was performed by unpaired two tailed *t*-test. ** $p < 0.01$ compared to PBS treated wounds at the same time point.
